# Supplementary figures and images for: Isolation of strains and their genome sequencing to analyze the mating system of Ophiocordyceps robertsii
Source: PLoS One. 2023 May 2;18(5):e0284978. doi: 10.1371/journal.pone.0284978 (PMC10153710; doi:10.1371/journal.pone.0284978)

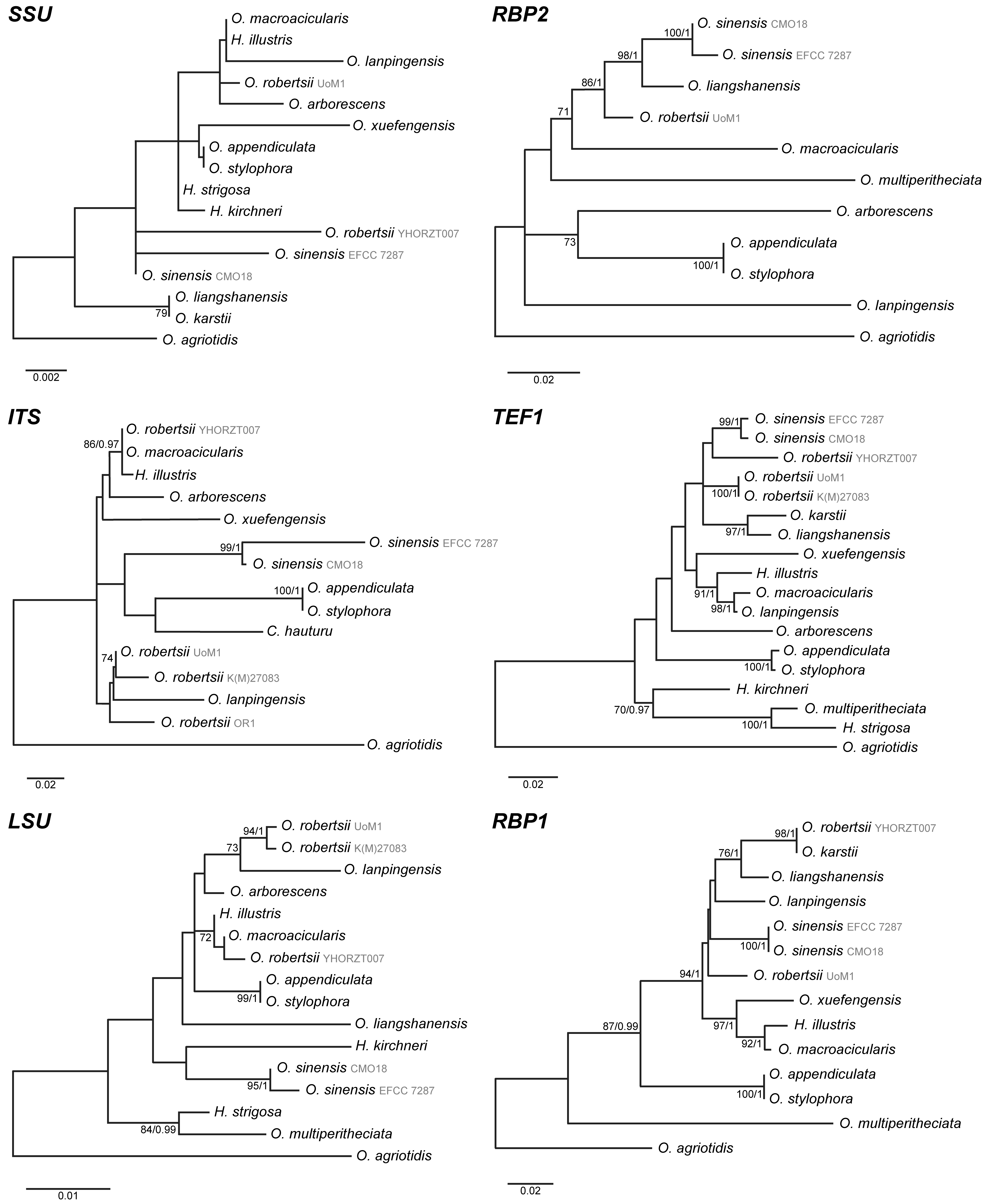

Supplement: S1 Fig — H is the abbreviation for Hirsutella and in the ITS tree C is the abbreviation for Cordyceps. O. agriotidis was used as the outgroup. Numbers adjacent to nodes indicate bootstrap % from 1000 replicates if above 70% / Bayesian posterior probability if above 0.95. Models used were Kimura-2 +G (SSU), Tamura-Nei +G (RBP2, TEF1), Hasegawa-Kishino-Yano +G (ITS, LSU) and Tamura-3 (RBP1). (TIF) [file pone.0284978.s001.tif]

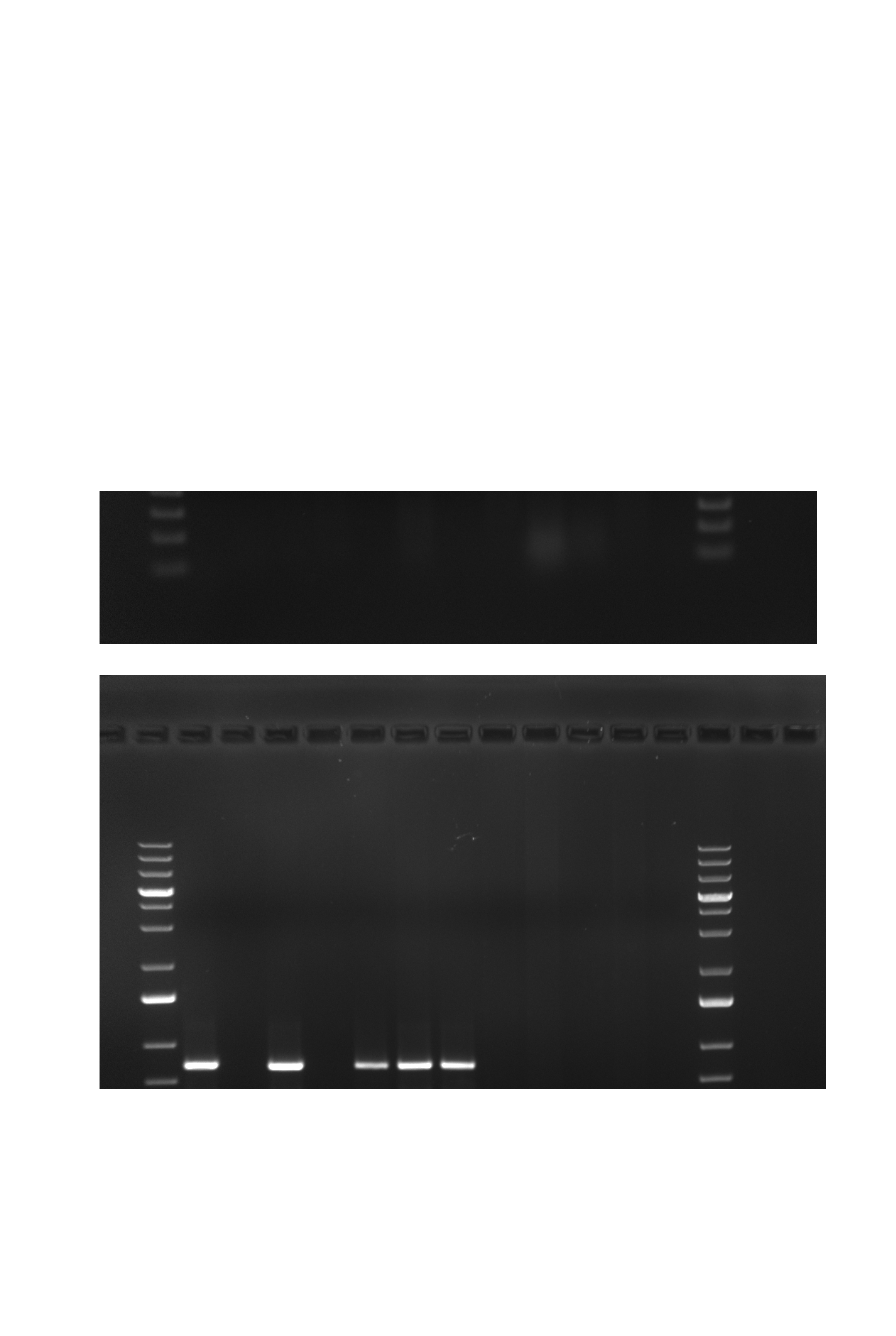

Supplement: S2 Fig — A. MAT1-1-1. B. MAT1-2-1. Both gels are loaded in a similar manner as follows. Nothing was loaded in wells 1, 16 and 17. A DNA ladder (sizes provided on the right) was loaded in wells 2 and 15. Amplification of progeny DNA was loaded into wells 3–14, with the PCR water control loaded in well 14. * indicates an additional amplification product. Images captured 27 April, 2020 using a Bio-Rad® gel documentation system. (TIF) [file pone.0284978.s002.tif]
