# Supplementary material for: Isolation of strains and their genome sequencing to analyze the mating system of Ophiocordyceps robertsii
Source: PLoS One. 2023 May 2;18(5):e0284978. doi: 10.1371/journal.pone.0284978 (PMC10153710; doi:10.1371/journal.pone.0284978)
Supplement: S3 Fig — The two strains of O. robertsii encode five predicted proteins. Best matches in GenBank or of relevance for genome arrangement are illustrated. H = Hirsutella, T = Tolypocladium and camp.-rufi. = camponoti-rufipedis. The … symbol indicates missing sequence information. (PDF) [file pone.0284978.s003.pdf]

MAT1-1-1

```
O. robertsii       MTTRNEVMQRLSSVRADVLLNFLTDDAIFQLASRYYESTTEADVLTPVSIAAPSRTTGQTKEASCDRAKRPLNAFMAFR
O. sinensis        MTTRNEVMQRLSSVRADVLLNFLTDDAIFQLASRYHESTTEADVLTPVSTAAASRATRQTKEASCDRAKRPLNAFMAFR
O. xuefengensis                                                         ...TTKQTKEASCDRAKRPLNAFMAFR
H. rhossiliensis   MTTRAEVLHHLSFVRSDILLNFLSDDAIFQLASRYYESTTEADVLTPVATAAAPRATGQTKDVSCDRAKRPLNAFMAFR
O. australis      MEATRAETLRRLSTAHREELLDLLSDEAINQLANRWEQTQTKIAVHNILVVNEATTVENLVQGTNSDRAKRPVNGYMAFR

O. robertsii      SYYLKLFPDVQQKTASGFLTTLWHKDPFRNKWALIAKVYSFVRDQIGKDKVSLAYFMSLACPAMTIIEPAAYLNTLGWFV
O. sinensis       SYYLKLFPDVQQKTASGFLTTLWHKDPFRNKWALIAKVYSFVRDQIGKDKVSLSYFMSLACPTMTIIEPAAYLNALGWCV
O. xuefengensis   SYYLKLFPDVQQKTASGFLTTLWHKDPFRNKWALIAKVYSFVRDQIGKDKVSLAYFMSLACPTMSIIEPAAYLNALGWFV
H. rhossiliensis  SYYLKLFPDVQQKTASGFLTTLWHKDPFRNKWALVAKVYSFVRDQIGKDKVSLAYFLSLACPIMSIIEPAAYLNALGWFI
O. australis      SYYRKIFPGVEQKFVSGFITILWHKDPFPNKWALIAKVYSFVRDQVGKSNATLSRFLEKACPQMQIIEPADYLDALGWSV

O. robertsii      QDDDAGSQKLFQDESSAN-LDQSSLLSAEYLSTEIELLSALVSAGYFPDQGADLVERMGSSQNGIMATRSAGYTPPVSYT
O. sinensis       QDDDAGSQKLFQDESSAN-LDQSSLLSAEYPSTEIELLSALVNIGYFPDHGADLVERMGSSHSGIMAPRAANCTPPVSYT
O. xuefengensis   QDD-AGSQKLLQDESSSK-LDQPGLLSAEYPSTEVELLSALVSNGYFPDQGVDLVERMGSSHSGIMATRAASFTPEVSYT
H. rhossiliensis  QEDDAGSKKLVQDESSAT-LDQSSSLSSEYPSTEIELLSTLVEVGYFPDQGVDLVERMGSSHSGIMATRAAKYALPVSYT
O. australis      HVNENGSLQLVRDESFANRYHSENEPSKAAPSTEMDLFKSILDSEFLNGMGAILLEKLSSNPNAVMAIRDANLG--SDMA

O. robertsii      KEKIDFINTIRTDPVQATKEILGDCYDETTIKLLGVKSHNVESVDSITHLSMQREYQDPRFFYDYSVSYAGMDFGGSNEP
O. sinensis       KEKIDFINTIRSDPVQATKEILGDCYDETTIKLLGVKSHNVESVDSITHLSMQREYQAPRFFYDYSVSYAGMDFGGSNEP
O. xuefengensis   KEKIDFMKTIRSDPVEAAKEILEDCYDETTIKLLGVKSHNVESVDSITHLSMQREYQHPRFFYDYSVSYAGMDLGGSNEP
H. rhossiliensis  KEKMGFIDMIRSDPIQASKDILGDCYNENTIRHFGVKSYNVENVDSITHLSMQREYQDPRSFYDYSVSYAGMDIDGSNEP
O. australis      MDQFELIDNLNAQSGQPVREVPAEGFDNERIDDGGAQTHHVANVDSILHLDMQQEYSDPVNSYDFGTSLYDFPFTDPSYN

O. robertsii      VMNLNDLPDNETFDIDSPFDLDKILGQSQSEGERTSHLPPSPPHNPLDDFHFAF
O. sinensis       VMNLNNLPENETFDIDSPFDLDKILGQSQSEGERTSHLPPSPPHNPLDDFYFAF
O. xuefengensis   VMNFDTLPENESFDIDSPFDLDKILGHSQSEGERTSHLPP
H. rhossiliensis  VMRFDNIPENETFDIDSPFDLDKILGQSQSEGERSE
O. australis      LFPCQNLFGIETYDVRDPSDFDRFMGYTQTEGEKSANLPPSRIYSPYEDFYRPI
```

MAT1-1-2

```
                                                                        <---- intron? ---->
O. robertsii      MESIISFEPFWKQSDVISTPRDALNDIRERSLNLFLDKHKGEPPLPS----SVEGKYFPALAFDHPLIKILTTAVVFECM
O. sinesis        MESICSFEPFWKQSDVVSAPRDALHDIRERSLNLFLDKHKGEPPLPS----SVEGKCFPVIVLDDPLMKILTTAVVFECM
O. xuefengensis   MESICSFEPFWKQTDVISAPQDAIQDIRVRSLNIYLEKHGEEPPLPS----SVQ-------------------ALVFECL
H. minnesotensis  MESICSFEPFWDLPDVISAPYEALHHIRTQALNTFLKNHRGKPRQPF----SVEN-------------------TIRECE
O. australis      MESICSFEPFWAHSDVIQSPKEAIDDIQKRVIYLYLNHYKNKKPEPLNIKD-----------------------AIKDCT

O. robertsii      EITRLLLEKLDDGNEILQRLHTIAVYHHNDAIYVVKGALAMWYSSAIPAVSRDPLQGLNPEALREFDENGDEFDPWSRRF
O. sinesis        EITRLLLKKLDDGNEILQRLHAIAFYHQNNAMYIVEGAFAMWYSSAIPAVSRDPLQGLNPEARREFDENGDEFDPWSRRF
O. xuefengensis   EITRLLLESPDDGNEILQRLHAIAFHHHNDAVYIVEGALAMWYSSAIPAISRDPLQGLNPEAPREFDGNGDEFDPWSRRF
H. minnesotensis  LVIQAILENTDDNNEVLQRLHDFGHHHQCGAFSIVQAALAMWYTSAAPVLARDPLQGLHPDSSSEVDESGRDYLPWSRSF
O. australis      AIIKALVKHPKDGNTILNRLSTTSGDFETATIDIVQGALAMWYTSIVPAVIRDEIQVCPAYLERRVNENGEPFLRWNREL

O. robertsii      ATQQHRAANLGIVAMLMTFKSRDSEAHPLSKAASLVARSVATLLYAVFLICPRMLEETWTHYLESTRPDETIKTFLRSTW
O. sinesis        ATQQHRAANLGIVAMLMTFKSRDSEAHPLSKTASLVARSVATLLYAVFLISPRMLEETWTHHLESTRPDETIKTFLRSTW
O. xuefengensis   ATQQHEAANLGIVAILMTFKSRYPEAHSLSKTASLVSRSVATLLYAVFLISPRMLEETWTHHLEVTRPDETINTFFRSTW
H. minnesotensis  SSKQHAAANLGIVAALMTSDRWIKQPHPLLDDAVLVSRSVTTLLYAVYLICPRMMDVDWTHQVELTRPMETIRTFLKSIW
O. australis      AREQLKNANLGLTAYFIAFGSWVPPQNPLLRTAILMSRSVSTILYAAFLLAPEMYHEDLKDLVRSTKPEETMGLYIKSIW

O. robertsii      SLTRENCTQLNVPAPGLEFGTTLDEVRLTRDGSNLITRIGR-FSWHSPPAWHPLRNVPGSPWNKFLRNHSQPIFPETPSQ
O. sinesis        SLTRENCTQLNVPAPGLEFGTTLDEVRLTRDGSDLITRIGR-FSWHSPPTWHPLRNVPGSPWNKFLRNHSQPIFPEAPSQ
O. xuefengensis   SLTRENCIGLNMPAPGLEFGASLDEVSLTSDGADLITRIGG-PSWQSPPAWHPLRNVPGSPWNKFLRNHSQPIFPETPSQ
H. minnesotensis  SLMRVGFDQMDAIPPGIEFGATLDEVTLSRNGMDIMTSIGS-QVWHSPPYWHPLRRVPGSAWNKFLRNHEQPIFPESQSQ
O. australis      SLAREDFDQFDASAPGAEFGATLDEVKLTRGGSKLLTNIGRGKSWYTPPYHHPLITMPGSPWGKFLRNHNRPFFTPPSSA

O. robertsii      SAGFRVTIPGSLRTLASPFEEYYSELRSRMDQTGRQRRVLHREAQIHQFHSLAEATGRRYPAMDLSEDNASYTEEPVQDF
O. sinesis        SSDFRITIPGSLSTLASALEEYYFELRSRMDQTGRQRRVLHREAQIRQFHSLAEATGRRYPAIDLSEDNASYTEEPVQDF
O. xuefengensis   SSEFRITIPGSLSTLAGSFEEYYFELRSRMDQTGRQRRVLHREAQLRQFHSLANATGRSYPAIDLSEDNASYTEEPVQDF
H. minnesotensis  SPGFRITIPGSLKTLSSAYQTYYFDLRSRMDQTGRARRVLTEEAMARQFHSLAAATGKPYPMMELTEDNASYTEEPEQDF
O. australis      DTSISFRWPSSLDTLVAPYKEIYTGIRERLDSRGIPRKVLSEEEQAQQFERLSKATGLEYPNMEISEDTAAAVEQPEQDY

O. robertsii      LYKLPMVKKPICDMSGHLTFPFMNTLVRAWRFQDEPDEVEPDFHIMTFER
O. sinesis        LYKLPMVKKPICDMSGHLTFPFMNTLVRAWRFQDEPDEVEPDFHIMTFER
O. xuefengensis   LYKLPMIKKPICDMVGHLTFPFMNTLVRAWRFQDEPDEVEPDFHIMTFER
H. minnesotensis  LYKLPMIKKPITDMRGYLTFPFMNTLVRAWRFRQEPDEVDPDFHIMTFER
O. australis      LHLLPTIKQPITDGRGHLTFSFANAALRAMRFSQDPDEVDPDFRILTIEHKGLGTQQPGPNETHQDPDEIEPDFQILSIG

O. robertsii
O. sinesis
O. xuefengensis
H. minnesotensis
O. australis      HNG
```

MAT1-1-3

```
O. robertsii      MMRPRANIPVQNGVADTLVTFVASETEGNVHVFLPDTLEMGLVETIAMNFSRRVQQPVKVFHDNWREKYRLCPLLPGVQA
O. sinensis              MPVQNGVEDTLVTFVASETEGNVHVFLSDTLEMGLVETIAMNFSRRVQQPVKVFHDNWREKYRLCPLLPGVQA
O. xuefengensis    MRPRVKIPGQDGVADTLITFVSSETEGNVHIFLTDTLEMEIVETIAMNFSRRVQQPVNVFHDSWREKYRLCPLLPGAQA
H. rhossiliensis         MPVQNGVEDTLVTFVASETEGNVHVFLSDTLEMGLVETIAMNFSRRVQQPVKVFHDNWREKYRLCPLLPGVQA
O. australis                                 MEVQAFVPASFDLEVVKKIAANFSRRVREPVKVFYDDKRHKYRLCPLPDGHQR

O. robertsii      NNITYGSLCFECDMSEPHVQETNSKDDAAEGKIGHIPRPRNKWILYRQYQAAIIRQDLGKITASEMSTMIANMWRQESEA
O. sinensis       NNITYGSLCFECDMSEPNVPET---------KMARIPRPRNKWILYRQYQAAIIRQDLGKITASEMSTMIANMWRQESKA
O. xuefengensis   DNITYGSLCFECDMSEPNAVDTNSKD--GEGKKGHIPRPRNKWILYRQYQAAIIRQDLGKITASEMSTMISNMWRQESEA
H. rhossiliensis  NNITYGSLCFECDSSEP-LPEMNSKDDVVDGKTGHIPRPRNKWILYRQYHAAIIKKEIGLITASEISTMISNMWRQASEA
O. australis      NISSYGIYCFEVDVEASQSPPAHEQATFG-GSKSHIPRPRNSWILYRQHKYAEIKDQYPGLTAPALSTIIASMWRNESTD

O. robertsii      EKAVWQQKAEEEDRLHKEKYPDYKYTTKKSPSKGN
O. sinensis       EKAVWKQKAEEEDRLHKEKYPDYKYTTRKSPSKGI
O. xuefengensis   EKAVWQQKAEEEDRLHKEKYPDYKYTTKKSPSKES
H. rhossiliensis  EKAIWQQKAEEEDRLHKEKYPDYKYTTKKSPSKGN
O. australis      DKAYIAKRAP------------TKGLKRRSDQMAN
```

MAT1-2-1

```
O. robertsii       MANPVNMNPNPQWNSTHYEAIWKSLEGQVNPFSQVLCLEGDFFRQLDDAAKLFIARKLMEHVQESVLYVNDGNGPDRVY
O. sinensis        MANPINMIPNPQWNATDYEAIWKGLEAQVNPFSQILCLEGDFFRQLDDAAKLFIARKLMEHVQESVLYVNDGNGPDRVY
O. xuefengensis    MANPINMNPNPQWNSTNYEAIWKGLEGQVNPFSQVLCLEGDFYRQLDAAAKLFLARKLMEHVQESVLYVNDGNGPDRVY
O. YHORZT007                                   ...PFSQVLCLEGDFYRQLDDTAKLFIARKLMEHVQESVLYVNDGNGPDRVY
O. lanpingensis                                ...PFSQVLCLEGDFYRQLDNAVKLFIARKFMEHVQESVLYVNDGNGPDRVY
T. paradoxum         MAANMNAATQWNPLQLQAIWRELEDQVHPFSHVLSIRGETFRQLDAAAKLFIARKFMEHVQESVLYVIDGNGVDRVY
O. camp.-rufi.    MDIPSNINNPGGLVTTPYNRQLWNRLQDQVHPFSQVLSIEGEIYRKLSPATKQYIAKKFMNHVQENVIFVVDGNGPDRAY
O. australis         MSFADNVAQQWDVLYLEALWSGLQGQINPFGQVLCLEGPVYRSLHDGARDYIANKFMQHIGQSVIYVLDGNGPDRVY

O. robertsii      LGAPRHFVVGGGMILQVSGYAPYWIRRS-VSKVITATVLAPPEP-KDIKIPRPPNAYILYRKERHHHVKDANPGITNNEI
O. sinensis       LGAPRHFVVGGGMILQISGYAPYWIRRS-VSKVVTATVLAPPSP-KDIKIPRPPNAYILYRKERHHYVKDANPGITNNEI
O. xuefengensis   LGAPRHFVVGGGMILQISGYNPYWIRRS-VSKVVTATILAPPEP-KDIKIPRPPNAYILYRKERHHQVKDANPGITNNEI
O. YHORZT007      LGAPRHFVVGGGMILQISGYAPYWIRRS-MSKVVTATILAPPEP-KDIKIPRPPNAYILYRKERHHQVKDENPGITNNEI
O. lanpingensis   LGAPRHFVVGGGMILQISGYNPYWIRRS-VSNVMTATVLAPPEP-KDVKIPRPPNAYILYRKERHHLVKEANPGITNNEI
T. paradoxum      LGAPKHLVDGGGTILRPPGNNLYWIRPA-VHKVTTATLIAPPAPPKHVKIPRPPNAYILYRKERHHHVKNSNPGITNNEI
O. camp.-rufi.    LGAPRHFTTGGGMVIYPSGRDLFWIRRG-QSNVMTATLMVPPLP-KKAKIPRPPNAYILYRKERHHMVKNANPGITNNEI
O. australis      LGAPRHFLAGSGKIFCLPGDKLYWTRDYGAHKINVDEPSSNPEPKKEATIRRPPNAYILYRKERHHLVKNSNPKITNNEI

O. robertsii      SQILGKAWNMESNDVRQKYKDMSQQVKQALLEKHPDYQYKPRRPCERRRRRRTSSNQNPKQSTSKDAATENAAISTGNTS
O. sinensis       SQILGKAWNMESNDVRQKYKDMSQQVKQALLEKHPDYQYKPRRPCERRRRRRASPNQNPKQSTSRNAATRDAAISSEDTS
O. xuefengensis   SQILGKAWNMESNEVRQKYKDMSQQVKQALLEKHPDYQYKPRRPCERRRRRRASPNENTKQSTSNNAAAKDAAISTGGTS
O. YHORZT007      SQILGKAWNMESNEVRQKYKDMSQQVK...
O. lanpingensis   SQILGKAWNMESNEVRQKYKDMSQQVK...
T. paradoxum      SQLLGKAWNLESREVRQKYKDMSEKVKQALLEKHPDYQYRPRRPSERRRRRRNAQSQDQQDNTATNNATTSPGDASAAAA
O. camp.-rufi.    SQLLGKAWNIESAEIRAKYKKMSDDIKEALMKKHPDYQYRPRRAGERRRRRRNDDQDEETPTANA
O. australis      SQILGKAWNLETPEIRLKYKIMSDDLKKALLEKHPDYQYRPRRPSERRRRRRNQKQPEDNDAGPDGASTSTADVVDGGSS

O. robertsii      TATGDSNTADGI
O. sinensis       TATGDTNTANGF
O. xuefengensis   TATDAPNIANGV
O. YHORZT007
O. lanpingensis
T. paradoxum      TPSDSTDTV
O. camp.-rufi.
O. australis      ANEDHFHGSTDFESAMNEFTNAF
```

MAT1-2-2

```
O. robertsii      MAN-TTPASLVPETTLQRLAPQREGIWFVTEQTGDHFGLAYVMDEGTHAINYQMFPCNTEEEAVEICGHLIYTYSGYDPV
O. sinensis       MAN-ANPASLVPMTTLQRLAPRREGIWFVTGQADDHFSLAYVMDEGTHALNYQMFPCNTEEEAVDICSHLIYTYSGYDPV
O. xuefengensis   MAN-ATPAGLVPETTLQRLAPRRDGIWFVTEQADDHFSLAYVMDEGTHALNYQIFPC...
T. paradoxum      MAN-VTPASLVPETTLQRLAPRREGFWLVAGQHTDHFTLAYVMDGGTHALTYHIYPCNTSEEVYDVCGHLMYDWTGAPDV
O. camp.-rufi.    MANPTSPDSLVPAAILQQLAPQEAGFWLIAEQALDHICLAYIMGRGTHAIAYRIATCSTVADVLSGYQHLIYSHP--DEQ
O. australis      MAD-ASPDNFVPEATLRRLAPPREGRWFVVDQNSSSFTLQYAMGSDTPAVSFEVVPCHTEQDIVDKLDGHLYKYTGYIPE

O. robertsii      NVPSPSHGRENAP---RPLHIVLRPRPQNPQGQGSVAQNTTGINQRYDPNQDHMAIGASLAAPEHCG----PAVQRSLEE
O. sinensis       NMPSPSHGRENAP---RPLHIVLRPGPQN---------NATGINQKYDPVQDHMAIGASLAAPEHCG----PAVQRSLEE
O. xuefengensis
T. paradoxum      IPSRFSYNRPLAVPGNQPLELALRPRDQDTQEQAHVPQNLLGTNLNLSSIQDQVAMVQQPAMAAQAHGQMDPAQDGAPQG
O. camp.-rufi.    TSGSVLHSPDQNCRFPQPVHIQLSGDIGHATDQPHDAHHNSGVHLDHATIRTQVLAALQTAEANPPQ-----NSLGTTDD
O. australis      NVSTPHTNGQVPP---LEISLTLRDDNGNPLPNEPQVPDEYVVSVNAAERTQQLLAAASYNFAPPVAE---------PQN

O. robertsii      IAYQFTPTDIHGLNQQQMPVPSIENGNGNLDWAMANDSLIDMTLMTDNQNSQTLDGLF---ERNTPLGSMFNDLSSTSGD
O. sinensis       IAYQFTPTDTHGLNQQQINVPSNENGNGHLDWAMANDSFNDMDLMADNQSSQTLDGLF---EINTPSGFMFNDLSSASGD
O. xuefengensis
T. paradoxum      TQYQLTQSMTHGQDQQQLINSADEHGN--IDWSMTNDLFNDITPMVNDQPDNPFAPLS---GDSATFASVLNEYSSTAGD
O. camp.-rufi.    FIHDNGAQPSMDGRSQEIPLNAT-FTNEEFDWTMDNEILRNTCLMFQEQVNFPLETFSGDVEEPSNLSVALDQSFMTEAE
O. australis      HQQDLTGNETGG------------------LYWNMTAQAFNEMSPDNNNEFN-----FHALVGDQGGLGNNFDNSMAIDQD

O. robertsii      MTLVNGENPEQVDWNIFHEG
O. sinensis       MTLVNGENQEQVDWNIFHEG
O. xuefengensis
T. paradoxum      MSLLDNDGQMQLEWNLFTDN
O. camp.-rufi.    PSVLDACSESQIDWNLYTTNN
O. australis      LTLGDAN-IDPVQWNAFQPIN
```
